# TREM 2 in Parkinson’s Disease: A Promising Candidate Gene for Disease Susceptibility and Progression

**DOI:** 10.3390/brainsci15040379

**Published:** 2025-04-05

**Authors:** Paolo Alonge, Carmela Rita Balistreri, Angelo Torrente, Daniele Magro, Elisa Rubino, Roberto Monastero

**Affiliations:** 1 Memory and Parkinson’s Disease Center, Department of Biomedicine, Neuroscience and Advanced Diagnostics (Bi.N.D.), University of Palermo, Via La Loggia 1, 90129 Palermo, Italy; paolo.alonge01@unipa.it (P.A.); angelo.torrente@unipa.it (A.T.); elisa.rubino@unipa.it (E.R.); 2 Cellular and Molecular Laboratory, Department of Biomedicine, Neuroscience and Advanced Diagnostics (Bi.N.D.), University of Palermo, 90134 Palermo, Italy; carmelarita.balistreri@unipa.it (C.R.B.); daniele.magro@unipa.it (D.M.)

**Keywords:** TREM2, PD, Parkinson’s disease, neuroinflammation, microglia, neurodegenerative disorders

## Abstract

**Background/Objectives**: The activation of microglia and the activity of innate immunity have recently been recognized as part of Parkinson’s Disease (PD) pathophysiology. Triggering receptor expressed on myeloid cells 2 (TREM2) is a gene with neuroprotective roles. Its variations are associated with microglial-associated neurodegeneration. The objective of the present review is to investigate the current evidence on the role of TREM2 in PD pathophysiology. **Methods**: A comprehensive search was performed using PubMed, Medline, and Web of Science, looking for English papers investigating the role of TREM2 in PD, or more in general, the genetic profile of microglia. **Results**: Thirty-one papers were considered relevant. Preclinical studies with PD models showed some contradictory results, even if a loss of function of TREM2 is generally associated with a microglial activation in α-synuclein-induced inflammatory processes. The role for TREM2 genetic variations in PD patients should be taken with even more caution. The increase in the soluble extracellular segment of TREM2 (sTREM2) in cerebrospinal fluid of PD patients seems to be associated with increased risk of cognitive decline. **Conclusions**: There is increasing evidence that TREM2 may have an important role in PD pathophysiology as demonstrated by preclinical and clinical studies. Further investigations are needed to confirm this role and may lead the way for future targeted therapies for different neurodegenerative disorders.

## 1. Introduction

Parkinson’s disease (PD) is a neurodegenerative disease that belongs to the synucleinopathies. The disease is characterized by the progressive loss of dopaminergic neurons in the substantia nigra (SN) and by the pathological accumulation of intraneuronal aggregates of alpha-synuclein (α-syn) [1]. The latter is considered as the potential diagnostic biomarker for PD, although definitive evidence is needed to consider it as a definitive biomarker for the disease [2]. Another crucial feature of PD is the activation of microglia and the activity of innate immunity, as consistently demonstrated in human studies of the brains of affected individuals using in vivo imaging techniques and post-mortem studies [3,4].

Microglia cells constitute the brain’s resident immune component and display a phenotype similar to that of mononuclear macrophages, with the fundamental role of protecting the central nervous system (CNS). Consequently, when activated, microglial cells evoke neuroinflammation and chronic neuronal damage, releasing inflammatory molecules (i.e., cytokines) and covertly diminishing their role in tissue surveillance [5]. This also causes changes in the interactions with synapses and prolongs the inflammatory response, leading to CNS damage. Furthermore, variations in immune response gene clusters, the expression of immunoglobulin family members, and several related microRNAs, as well as the expression of NF-κB, and pro-inflammatory cytokines and apoptotic biomarkers in human microglia and mouse brain supported this evidence. Precisely, it has recently been shown that variations in the activation of the triggering receptor expressed on myeloid cells 2 (TREM2), one of several genes whose expression is restricted to microglia in the brain, increase the risk of developing PD [6]. This causes microglia cells to progressively acquire a unique transcriptional and functional signature and evolve into disease-associated microglia (DAM).

TREM2 is a member of the immunoglobulin superfamily and precisely a transmembrane protein expressed by myeloid cells, such as microglia [7]. TREM2 is critical for preserving the metabolic fitness of microglia during stress events, supporting the progression of microglia towards a fully mature DAM profile, and ultimately supporting the transition of microglia from an anti-inflammatory (M2) to a pro-inflammatory (M1) phenotype. The M1 phenotype is induced by lipopolysaccharide (LPS) or interferon-gamma (IFN-γ), resulting in the production of pro-inflammatory mediators such as tumor necrosis factor-alpha (TNF-α), interleukin-1 beta (IL-1β), and reactive oxygen species (ROS), which contribute to neurotoxicity and chronic neuroinflammation in conditions such as Alzheimer’s disease (AD) and PD [8,9]. In contrast, the M2 phenotype, driven by interleukin-4 (IL-4) or interleukin-13 (IL-13), upregulates anti-inflammatory factors like interleukin-10 (IL-10), transforming growth factor-beta (TGF-β), and arginase-1 (Arg-1), promoting tissue repair, phagocytosis of debris, and the resolution of inflammation [8,10]. Although emerging evidence suggests that microglia exist along a spectrum of activation states in vivo, targeting microglial polarization by enhancing M2-associated pathways or suppressing M1-driven neuroinflammation, is a promising strategy under evaluation for neurodegenerative and neuroinflammatory diseases [8]. These changes enhance the removal of apoptotic neurons and attenuate inflammation-induced neurodegenerative phenomena [11]. These crucial roles of TREM2 and its changes in gene expression, as well as its genetic variants, have gained significant attention from neurodegenerative disease researchers.

Genetic variants of TREM2 have been implicated in elevating the risk of developing AD [12]. In particular, TREM2 appears to play a critical role as a microglial receptor for amyloid-beta, facilitating its phagocytosis and clearance, thus contributing to the regulation of amyloid pathology in AD [13]. Furthermore, TREM2 mutations have been implicated in other neurodegenerative diseases, such as frontotemporal dementia and progressive supranuclear palsy [14,15].

Given the crucial role of TREM2 in microglial activation, the relationship between PD and TREM2 activity has been the subject of increasing research interest in recent years, although results have been somewhat inconclusive. In this narrative review, we sought to provide a comprehensive overview of the current evidence on the role of TREM2 in PD from clinical and preclinical studies. Our aim is to clarify the existing knowledge and propose directions for future research.

## 2. Materials and Methods

For this review, a comprehensive search was performed using the following international databases: PubMed, Medline, and Web of Science. The search (limited to articles in English language, without any restriction on ethnicity or geographical area) included the following keywords: ‘TREM2’ and ‘PD’, ‘TREM2’ and ‘Parkinson’, ‘genetic profile of microglia’ and ‘PD’, ‘genetic profile of microglia’ and ‘Parkinson’. First, the titles were checked to exclude double matches, off-topic, or non-English papers. The remaining abstracts and, afterwards, full texts were examined to judge the relevance of the papers to the topic of the review. Specifically, our inclusion criteria included both clinical and preclinical original studies that investigated the role of TREM2 in Parkinson’s disease (PD) across multiple dimensions, including pathophysiology, potential diagnostic and/or prognostic biomarkers, and therapeutic targets.

## 3. Results

A total of 711 results were retrieved. After the examination, 31 papers were considered relevant and therefore included in the review. For clarity, the preclinical and clinical studies were discussed separately. Preclinical studies, which included in vitro and animal models, explored the molecular mechanisms and the pathophysiological role of TREM2 in PD under controlled experimental conditions, while clinical studies evaluate the translational relevance of these findings in the human population, examining associations with disease progression, biomarker utility, and therapeutic applications. Given the distinct methodologies and research objectives of these two approaches, splitting them allows for a more systematic and consistent analysis of the role of TREM2 in PD.

### 3.1. Preclinical Studies

Preclinical studies are summarized in Table 1. Given the diverse methodologies employed, the studies were further categorized based on the disease model utilized.

#### 3.1.1. MPTP-Mice Models

The function of TREM2 has been extensively studied in one of the most widely recognized and used mouse models of PD, the 1-methyl-4-phenyl-1,2,3,6-tetrahydropyridine (MPTP)-induced model [16]. TREM2 appears to be closely associated with autophagy processes, which are critically involved in the pathogenesis of PD. Autophagy is a key mechanism for the removal of α-syn aggregates and damaged mitochondria, both of which are implicated in PD pathology [17]. Impairment of these processes is a hallmark of PD progression, highlighting the potential importance of TREM2 in disease mechanisms. Increasing TREM2 levels raise autophagy in microglia, mediated by the mTOR pathway, as demonstrated by in vitro and in vivo studies. TREM2 levels in microglia can be increased by administering 18β-glycyrrhetinic acid, the main active metabolite of glycyrrhizic acid, known for its neuroprotective effects [18]. This activity appears to be mediated by TREM2 through the induction of the M2 phenotype in microglia, which promotes the expression of the brain-derived neurotrophic factor (BDNF), a key factor in promoting the survival of dopaminergic neurons [19].

Regarding the regulation of inflammation, Belloli et al. found that TREM2 knockout mice exhibited reduced levels of pro-inflammatory cytokines, with lower inflammatory response following MPTP exposure; however, this did not confer protection against the loss of dopaminergic neurons, suggesting the involvement of more complex inflammatory mechanisms in acute neuronal injury [20]. Conversely, Ren et al. demonstrated that TREM2 overexpression attenuates neurodegeneration and neuroinflammation [21].

Furthermore, the repopulation of microglia after temporary depletion in MPTP mouse models increased TREM2 transcript levels and reduced MPTP-induced toxicity, thus slowing the development and progression of neurodegeneration [22]. Interestingly, the suppression of DJ-1 expression, an oxidative stress sensor located in the mitochondria, reduced TREM2 expression, suggesting that oxidative stress may induce a shift in microglial activity towards the M1 pro-inflammatory phenotype. Furthermore, pretreatment of microglia with rasagiline, a monoamine oxidase inhibitor, reduces neurotoxicity, indicating that PD medications may exert protective effects independently of their direct actions on neurons [23].

#### 3.1.2. Other Animal Models

**Figure 1:**
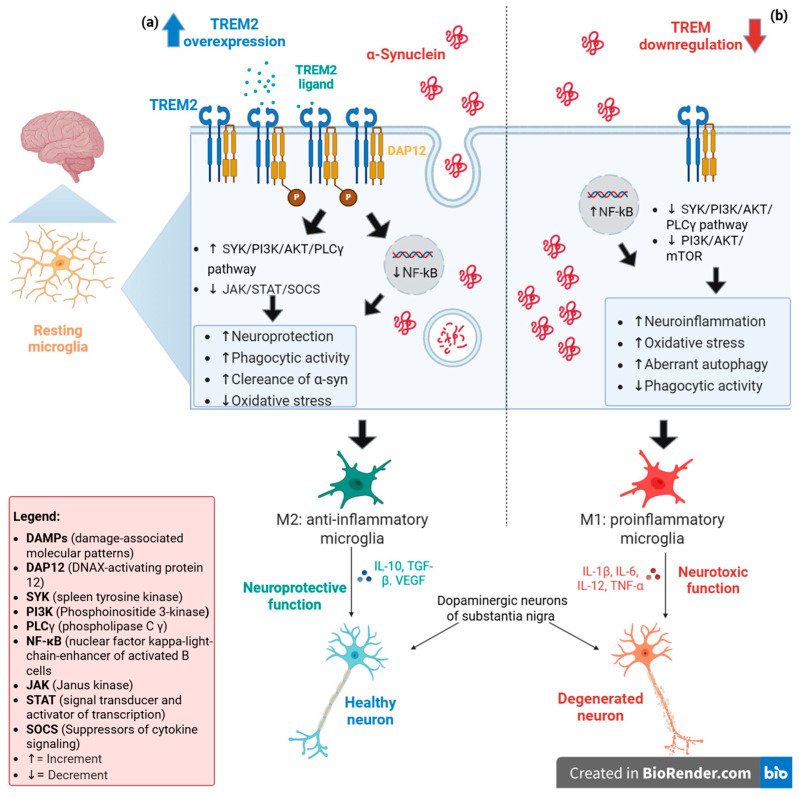
TREM2 expression levels could induce two distinct phenotypes in resting microglia. (**a**) In a scenario where TREM2 is overexpressed, its activation, mediated by one of its potential ligands (DAMPs or lipid components), promotes the phosphorylation of DAP12, a TREM2-associated transmembrane adaptor protein. This phosphorylation generates a docking site for many molecules, triggering signaling cascades that lead to the activation of SYK/PI3K/AKT/PLCγ pathway, which potentiates phagocytic activity and the regulation of PI3K/NF-κB and JAK/STAT/SOCS pathways allowing the anti-inflammatory effects. The combination of these events drives the cell toward an M2 phenotype, which has a neuroprotective function for neurons and ensures the elimination of α-synuclein aggregates [31,32]. (**b**) In contrast, a reduced TREM2 expression promotes the shift in microglia towards the M1 pro-inflammatory phenotype due to the lack of DAP12 phosphorylation, which causes deregulation of PI3K/NF-κB and JAK/STAT/SOCS pathways (favoring inflammatory processes) and decreased PI3K/AKT/mTOR and SYK/PI3K/AKT/PLCγ activation, leading to aberrant autophagy episodes. The final effect of these processes could damage the neurons that undergo accumulation of α-synuclein aggregates due to deregulation of phagocytosis [13,32].

The analysis of the TREM2 function in other animal models of PD has yielded contradictory results. In synucleinopathy models, both in vivo and in vitro studies showed that TREM2 deficiency increases microglia activation during α-syn-induced inflammatory processes, leading to an increased loss of dopaminergic neurons (53% vs. 28% after 3 weeks) in animal models [24]. Furthermore, as also seen in MPTP-induced models, the absence of TREM2 exacerbates microgliosis and astrocytosis in the basal ganglia during chronic inflammation, effects due to its influence on the TLR4/MyD88/NF-κB pathway, whereas its overexpression increases autophagy via the PI3K/AKT/mTOR pathway [25,26]. Moreover, α-syn monomers induce an upregulation of TREM2 expression, proposing a possible mechanism to explain why PD patients show increased levels of TREM2 [27]. Figure 1 provides a summary of the TREM2-mediated mechanisms that regulate microglial activation via the PI3K/AKT/mTOR pathway.

In 6-hydroxydopamine (6-OHDA) mouse models, dihydroquercetin, which showed neuroprotective activity in PD, increases TREM2 and reduces the loss of dopaminergic neuron in mouse models, but not in TREM2 knockout mice [28]. In the same mouse models, Liang et al. showed that TREM2 activates the TGF-β pathway to enhance neuronal repair and induces the differentiation of induced pluripotent stem cells (iPSCs) into dopaminergic neurons in the SN, thus demonstrating a possible role of TREM2 outside of the regulation of microglial activity [29].

In contrast, in NF-κB/c-Rel (c-Rel^−/−^)-deficient mice, which spontaneously develop a late-onset PD-like phenotype, TREM2 levels increase with age both in c-Rel^−/−^ and wild-type mice, thus demonstrating no specific role of TREM2 in neurodegeneration [30]. However, the c-Rel^−/−^ model does not induce pronounced microglia activation, possibly explaining the different findings with other models, but suggesting that other inflammatory and neurodegenerative mechanisms may be involved in PD pathology.

**Table 1:** Preclinical studies on TREM2 involvement in PD.

	Animal Model	Main Finding
Luo et al. [18]	MPTP-induced	18β-glycyrrhetinic acid reduces MPTP-induced toxicity by upregulating TREM2.
Belloli et al. [20]	MPTP-induced	Knocking out TREM2 reduces the levels of pro-inflammatory cytokines (i.e., IL-1β, TNF-α) and neuroinflammation; however, it does not confer protection against dopaminergic neuronal loss
Ren et al. [21]	MPTP-induced	TREM2 overexpression attenuates neurodegeneration and neuroinflammation by regulating the TLR4/MyD88/NF-κB pathway
Li et al. [22]	MPTP-induced	Repopulation of microglia after temporary depletion increases TREM2 transcript levels and reduces MPTP-induced toxicity.
Trudler et al. [23]	MPTP-induced	Suppressing the expression of DJ-1 in mitochondria reduces TREM2 expression, suggesting that oxidative stress may induce a shift in microglial activity toward a pro-inflammatory phenotype. Pre-treatment of microglia with rasagiline reduces neurotoxicity, indicating that PD medications may exert protective effects.
Guo et al. [24]	α-syn-induced	TREM2 deficiency increases the activation of microglia, leading to an augmented loss of dopaminergic neurons.
Huang et al. [26]	MPTP-induced	TREM2 knockdown Promotes NLRP3 inflammasome activation and inflammatory response, aggravating dopaminergic neuron loss.
Yin et al. [27]	α-syn-induced	Knocking out TREM2 worsens pathological α-syn spread, increasing dopaminergic neuronal loss. Reducing TREM2 levels impairs microglial phagocytosis and proliferation, but enhances autophagy via the PI3K/AKT/mTOR pathway. TREM2 overexpression enhances microglial responsiveness in the pathological site.
Yang et al. [28]	6-hydroxydopamine mice model	Dihydroquercetin increases TREM2 and reduces dopaminergic neuronal loss in mice models, but not in TREM2 knockout mice
Liang et al. [29]	6-hydroxydopamine mice model	TREM2 activates the TGF-β pathway, enhancing neuronal repair in the substantia nigra, and inducing the differentiation of induced pluripotent stem cells into dopaminergic neurons.
Porrini et al. [30]	c-Rel^−/−^	TREM2 levels increase with age both in c-Rel^−/−^ and wild-type mice, thus demonstrating no specific role of TREM2 in neurodegeneration in this model.

### 3.2. Clinical Studies

The TREM2 variant rs75392628 (c.234G>A) leads to the missense substitution of arginine with histidine (R47H). In 2013, Rayaprolu et al. linked this variant to an increased risk of PD (OR = 2.67) [14]. However, subsequent population studies failed to replicate these results [33,34,35]. Therefore, the actual relationship of the rs75392628 variant with PD is still debated. However, although the association between genetic variations in TREM2 and PD remains uncertain, a growing body of evidence from multiple studies points to the potential utility of TREM2 as a biomarker in clinical practice.

Soluble TREM2, the extracellular segment of TREM2 (sTREM2) released after proteolytic cleavage, showed higher levels in the cerebrospinal fluid (CSF) of PD patients compared to healthy controls, especially in patients with sleep disorders, with a positive correlation with α-syn levels [36,37]. In contrast, Qin et al. observed no significant differences in CSF sTREM2 levels between PD patients and healthy controls. However, they identified that elevated baseline CSF sTREM2 levels were predictive of a greater global cognitive decline and increased risk of cognitive impairment in PD subjects [38]. In peripheral blood samples, sTREM2 did not discriminate between PD and healthy controls; however, monocyte TREM2 was higher in PD patients at a high risk for dementia than in healthy controls, confirming the involvement of innate immunity in PD and suggesting a possible immunological link between PD at high risk for dementia and AD [39].

Another study that assessed levels of soluble TREM2 (sTREM2) in the CSF of both PD patients and healthy controls found no significant differences. However, basal levels of sTREM2 could predict motor progression over the four years of follow-up in PD patients; furthermore, the rate of change in sTREM2 in the CSF correlated with the progressive deterioration of motor function in PD individuals. Furthermore, patients with late-onset PD had higher levels of sTREM2 in the CSF than patients with early-onset PD [40].

Current evidence therefore suggests that TREM2 levels in CSF or peripheral blood, although not sensitive enough to serve as a diagnostic biomarker, can predict the risk of early cognitive involvement and dementia in PD patients, and even the risk of motor progression. Table 2 summarizes the clinical studies regarding TREM2 involvement in PD patients.

## 4. Discussion

In recent years, the interest in the role of TREM2 in PD has increased, given the growing recognition of the involvement of microglia in neurodegenerative disorders. Microglia-mediated neuroinflammation is a key component of PD pathophysiology, and an investigation of proteins that regulate microglia function, such as TREM2, could provide valuable insights into new therapeutic strategies, as well as potential diagnostic and prognostic biomarkers. Preclinical studies have greatly advanced our understanding of TREM2 in PD, highlighting its role in mediating microglial activation. However, preclinical studies revealed some discrepancies. Although variations in experimental models (e.g., the use of less commonly used models, such as the 6-hydroxydopamine mice and c-Rel^−/−^ mice) may contribute to these inconsistencies, some differences persist even among studies employing the widely used MPTP-induced PD model. Although research into TREM2 depletion and overexpression has not provided a conclusive explanation for its role in PD, it consistently implicates TREM2 in the regulation of microglial activity. Notably, its effects appear to vary in acute, subacute, and chronic inflammatory phases, as well as by brain region. These spatiotemporal differences, combined with interactions with other microglial regulatory mechanisms, may underlie the observed inconsistencies.

However, despite these findings, clinical studies have not yet provided conclusive evidence linking genetic variations in TREM2 to PD susceptibility, as has been the case of AD. In fact, while the R47H variant of TREM2 was initially associated with PD, subsequent studies have largely failed to replicate this finding. These inconsistent associations across neurodegenerative disorders may reflect emerging evidence of region-specific microglial modulation mediated by TREM2. Notably, a recent transcriptomic analysis of an R47H-TREM2 animal model revealed spatiotemporal regulation of TREM2 activity, demonstrating its role in modulating regional microglial density and activation states. This observed regional specificity may explain the differential association of TREM2 variants (including R47H) with various neurodegenerative disorders [42]. Although these findings provide important mechanistic insights, it should be noted that the study focused specifically on AD. Similar studies in animal models of PD could help understand the conflicting results from genetic studies of TREM2 variants in PD, while clarifying whether comparable region-specific analogous mechanisms influence the role of TREM2 in disease pathogenesis. Furthermore, emerging clinical studies have identified novel TREM2 variants whose potential association with PD remains unclear and warrants investigation in larger cohorts [37].

Regarding the potential clinical applications of sTREM2, current evidence shows consistently elevated CSF levels in PD patients across most studies, with the notable exception of Qin et al. (2022) [38]. One possible explanation for this discrepancy could be the inclusion of patients with an early diagnosis of PD (within 2 years) in the cited study, while other studies included patients with different disease durations. However, the utility of sTREM2 as a diagnostic biomarker is limited by its lack of disease specificity, as similar elevations are observed in multiple sclerosis and other neurodegenerative conditions [43,44]. However, its combination with other biomarkers, such as glial fibrillary acid protein GFAP, Myelin basic protein (MBP), and interleukins, could provide disease-specific signatures to discriminate between neurological disorders in the future—a currently unmet need in the real-world clinical setting.

However, sTREM2 has shown promise as a prognostic biomarker, with CSF levels associated with early cognitive impairment in PD. In addition, longitudinal evaluations suggest that sTREM2 levels may help predict motor progression over time. Although further studies are needed to clarify the exact clinical relevance of TREM2 in PD, current evidence points to its potential role in disease progression. If confirmed, such evidence could support the use of sTREM2 as a surrogate biomarker in clinical trials evaluating immunomodulatory treatments [31,41]. Moreover, preclinical research continues to shed light on its mechanistic involvement, raising the possibility of targeting TREM2 for therapeutic intervention, as has been explored in AD through multiple approaches: for example, dehydroervatamine has recently been demonstrated as a promising new TREM2 agonist that can attenuate neuroinflammation, [45] while TREM2 agonistic monoclonal antibodies are currently under development [46]. This, however, represents only the beginning of a long, but feasible, path due to the advancement of omics technologies [47].

## 5. Conclusions

The present overview reports TREM2 as an important modulator of microglial function in PD. However, several aspects remain unclear and will represent the subject of further investigations. Consequently, the crucial function of TREM2 in neuroinflammation is still debated. Further studies are essential to understand the involved mechanisms, as well as the endogenous stimuli and ligands that mutually determine changes in TREM2 expression, activation, or suppression. Their identification could provide new targets for the development of potential therapeutic strategies, such as the use of TREM2 agonists to alleviate neuroinflammation in microglia cells and treat CNS disorders associated with M1 activation and polarization of microglia cells. If confirmed, these data will have significant implications in clinical and prognostic settings for Parkinson’s disease.

## Figures and Tables

**Table 2:** Clinical studies on TREM2 involvement in PD. Note: Parkinson’s disease (PD); multiple system atrophy (MSA); frontotemporal dementia (FTD); amyotrophic lateral sclerosis (ALS); progressive supranuclear palsy (PSP).

	Study Population	Main Finding	Comment
Rayaprolu et al. [14]	609 patients with FTD, 765 with ALS, 1493 with PD, 772 with PSP, 448 with ischemic stroke, and 1957 healthy controls	TREM2 p.R47H variant increases susceptibility to FTD (OR = 5.06) and PD (OR = 2.67).	Strength: First large-scale study establishing a link between TREM2 variants and PD. Limitation: Did not adjust for potential confounders (e.g., comorbidities, environmental influences).
Chen et al. [33]	1216 patients with PD, 406 with MSA, and 869 healthy controls	TREM2 p.R47H variant showed no evidence of association to PD or MSA.	Strength: First TREM2 genetic study to include MSA patients. Limitation: Underpowered for rare variants due to limited sample size.
Dardiotis et al. [34]	358 patients with PD and 358 healthy controls	TREM2 p.R47H variant showed no association to PD.	Limitation: Smaller sample size compared to similar studies, reducing statistical reliability.
Mengel et al. [35]	821 patients with PD, of which 261 with dementia, and 919 healthy controls	TREM2 p.R47H variant showed no association to PD, even in the demented subpopulation.	Strength: Focused on cognitive decline in PD, a clinically relevant subgroup. Limitation: Small sample size for subgroup analyses.
Mo et al. [36]	80 patients with sporadic PD and 65 healthy controls	Soluble TREM2 levels are higher in the cerebrospinal fluid of PD patients compared to healthy controls, especially in those patients with sleep disorders.	Strength: Explored clinical correlations of TREM2 levels, including the incidence of sleep disorders. Limitation: Very limited sample, requiring validation in larger studies.
Peng et al. [37]	612 patients with PD and 680 healthy controls	Two novel TREM2 variants were identified (p.S81N, p.G58D), but they were not associated with PD. Soluble TREM2 levels were higher in the cerebrospinal fluid of a partial group of PD patients (n = 55) compared to controls (n = 40), but not in plasma.	Strength: Combined genetic and biochemical analyses, identifying novel TREM2 variants. Limitation: CSF data available only in a small subset, limiting generalizability.
Qin et al. [38]	219 PD patients and 100 healthy controls	Soluble TREM2 did not differ in cerebrospinal fluid among PD and healthy controls. Higher baseline levels of soluble TREM2 predicted cognitive decline risk in PD patients.	Strength: Longitudinal design revealed TREM2’s prognostic value for cognitive decline. Limitation: Predictive analyses may be underpowered considering the sample size.
Wijeyekoon et al. [39]	95 PD patients and age and sex-matched controls	TREM2 expression on monocyte was increased in PD patients at high risk for dementia. Soluble TREM2 in peripheral blood did not discriminate among PD cases and controls.	Strength: Innovative cellular approach (monocyte TREM2 expression). Limitation: Small cross-sectional sample without longitudinal follow-up.
Zhang et al. [40]	217 PD patients and 102 healthy controls	Soluble TREM2 levels in cerebrospinal fluid did not discriminate among PD patients and healthy controls. The change rate of soluble TREM2 was correlated with the progressive deterioration of motor function in PD patients.	Strength: Demonstrated that dynamic changes in TREM2 correlate with motor progression. Limitation: Requires replication in independent cohorts.
Pagan et al. [41]	75 PD patients	A single dose of nilotinib significantly increases the levels of TREM2 in cerebrospinal fluid, suggesting an anti- inflammatory effect.	Strength: The first to test the reliability of TREM2 as a surrogate marker of immunomodulation. Limitation: Pilot study with small sample size and without blinding.

## Abbreviations

The following abbreviations are used in this manuscript:

PD	Parkinson’s disease
SN	Substantia nigra
α-syn	Alpha-synuclein
TREM2	Triggering receptor expressed on myeloid cells 2
CNS	Central nervous system
DAM	Disease-associated microglia
LPS	Lipopolysaccharide
IFN-γ	Interferon-gamma
TNF-α	Tumor necrosis factor-alpha
IL-1β	Interleukin-1 beta
ROS	Reactive oxygen species
AD	Alzheimer’s disease
IL-4	Interleukin-4
IL-13	Interleukin-13
IL-10	Interleukin-10
TGF-β	Transforming growth factor-beta
Arg-1	Arginase-1
MPTP	1-methyl-4-phenyl-1,2,3,6-tetrahydropyridine
BDNF	Brain-derived neurotrophic factor
sTREM2	Soluble TREM2
CSF	Cerebrospinal fluid
TLA	Three letter acronym
LD	Linear dichroism

## References

[1] Nicoletti A., Baschi R., Cicero C.E., Iacono S., Re V.L., Luca A., Schirò G., Monastero R., Gender Neurology Study Group of the Italian Society of Neurology (2023). Sex and gender differences in Alzheimer’s disease, Parkinson’s disease, and Amyotrophic Lateral Sclerosis: A narrative review. Mech. Ageing Dev..

[2] Ganguly U., Singh S., Pal S., Prasad S., Agrawal B.K., Saini R.V., Chakrabarti S. (2021). Alpha-Synuclein as a Biomarker of Parkinson’s Disease: Good, but Not Good Enough. Front. Aging Neurosci..

[3] Trainor A.R., MacDonald D.S., Penney J. (2024). Microglia: Roles and genetic risk in Parkinson’s disease. Front. Neurosci..

[4] Samant R.R., Standaert D.G., Harms A.S. (2024). The emerging role of disease-associated microglia in Parkinson’s disease. Front. Cell. Neurosci..

[5] Balistreri C.R., Monastero R. (2023). Neuroinflammation and Neurodegenerative Diseases: How Much Do We Still Not Know?. Brain Sci..

[6] Mirarchi A., Albi E., Arcuri C. (2024). Microglia Signatures: A Cause or Consequence of Microglia-Related Brain Disorders?. Int. J. Mol. Sci..

[7] Pocock J., Vasilopoulou F., Svensson E., Cosker K. (2024). Microglia and TREM2. Neuropharmacology.

[8] Qin J., Ma Z., Chen X., Shu S. (2023). Microglia activation in central nervous system disorders: A review of recent mechanistic investigations and development efforts. Front. Neurol..

[9] Timofeeva A.V., Akhmetzyanova E.R., Rizvanov A.A., Mukhamedshina Y.O. (2024). Interaction of microglia with the microenvironment in spinal cord injury. Neuroscience.

[10] Lannes N., Eppler E., Etemad S., Yotovski P., Filgueira L. (2017). Microglia at center stage: A comprehensive review about the versatile and unique residential macrophages of the central nervous system. Oncotarget.

[11] Colonna M., Butovsky O. (2017). Microglia Function in the Central Nervous System During Health and Neurodegeneration. Annu. Rev. Immunol..

[12] Ulland T.K., Colonna M. (2018). TREM2—A key player in microglial biology and Alzheimer disease. Nat. Rev. Neurol..

[13] Hou J., Chen Y., Grajales-Reyes G., Colonna M. (2022). TREM2 dependent and independent functions of microglia in Alzheimer’s disease. Mol. Neurodegener..

[14] Rayaprolu S., Mullen B., Baker M., Lynch T., Finger E., Seeley W.W., Hatanpaa K.J., Lomen-Hoerth C., Kertesz A., Bigio E.H. (2013). TREM2 in neurodegeneration: Evidence for association of the p.R47H variant with frontotemporal dementia and Parkinson’s disease. Mol. Neurodegener..

[15] Sánchez-Ruiz de Gordoa J., Erro M.E., Vicuña-Urriza J., Zelaya M.V., Tellechea P., Acha B., Zueco S., Urdánoz-Casado A., Roldán M., Blanco-Luquin I. (2020). Microglia-Related Gene Triggering Receptor Expressed in Myeloid Cells 2 (TREM2) Is Upregulated in the Substantia Nigra of Progressive Supranuclear Palsy. Mov. Disord..

[16] Lal R., Chopra K. (2024). Experimental models of Parkinson’s disease: Challenges and Opportunities. Eur. J. Pharmacol..

[17] Luoma P., Melberg A., Rinne J.O., Kaukonen J.A., Nupponen N.N., Chalmers R.M., Oldfors A., Rautakorpi I., Peltonen L., Majamaa K. (2004). Parkinsonism, premature menopause, and mitochondrial DNA polymerase gamma mutations: Clinical and molecular genetic study. Lancet.

[18] Luo H., Zhang C., He L., Lin Z., Zhang J.-C., Qi Q., Chen J.-X., Yao W. (2023). 18β-glycyrrhetinic acid ameliorates MPTP-induced neurotoxicity in mice through activation of microglial anti-inflammatory phenotype. Psychopharmacology.

[19] Baydyuk M., Xu B. (2014). BDNF signaling and survival of striatal neurons. Front. Cell. Neurosci..

[20] Belloli S., Pannese M., Buonsanti C., Maiorino C., Di Grigoli G., Carpinelli A., Monterisi C., Moresco R.M., Panina-Bordignon P. (2017). Early upregulation of 18-kDa translocator protein in response to acute neurodegenerative damage in TREM2-deficient mice. Neurobiol. Aging.

[21] Ren M., Guo Y., Wei X., Yan S., Qin Y., Zhang X., Jiang F., Lou H. (2018). TREM2 overexpression attenuates neuroinflammation and protects dopaminergic neurons in experimental models of Parkinson’s disease. Exp. Neurol..

[22] Li Q., Shen C., Liu Z., Ma Y., Wang J., Dong H., Zhang X., Wang Z., Yu M., Ci L. (2021). Partial depletion and repopulation of microglia have different effects in the acute MPTP mouse model of Parkinson’s disease. Cell Prolif..

[23] Trudler D., Weinreb O., Mandel S.A., Youdim M.B.H., Frenkel D. (2014). DJ-1 deficiency triggers microglia sensitivity to dopamine toward a pro-inflammatory phenotype that is attenuated by rasagiline. J. Neurochem..

[24] Guo Y., Wei X., Yan H., Qin Y., Yan S., Liu J., Zhao Y., Jiang F., Lou H. (2019). TREM2 deficiency aggravates α-synuclein-induced neurodegeneration and neuroinflammation in Parkinson’s disease models. FASEB J..

[25] Balistreri C.R., Colonna-Romano G., Lio D., Candore G., Caruso C. (2009). TLR4 polymorphisms and ageing: Implications for the pathophysiology of age-related diseases. J. Clin. Immunol..

[26] Huang P., Zhang Z., Zhang P., Feng J., Xie J., Zheng Y., Liang X., Zhu B., Chen Z., Feng S. (2024). TREM2 Deficiency Aggravates NLRP3 Inflammasome Activation and Pyroptosis in MPTP-Induced Parkinson’s Disease Mice and LPS-Induced BV2 Cells. Mol. Neurobiol..

[27] Yin S., Chi X., Wan F., Li Y., Zhou Q., Kou L., Sun Y., Wu J., Zou W., Wang Y. (2024). TREM2 signaling in Parkinson’s disease: Regulation of microglial function and α-synuclein pathology. Int. Immunopharmacol..

[28] Yang R., Li D.D., Li X.X., Yang X.X., Gao H.M., Zhang F. (2024). Dihydroquercetin alleviates dopamine neuron loss via regulating TREM2 activation. Int. J. Biol. Macromol..

[29] Liang H., Liu P., Wang Z., Xiong H., Yin C., Zhao D., Wu C., Chen L. (2024). TREM2 gene induces differentiation of induced pluripotent stem cells into dopaminergic neurons and promotes neuronal repair via TGF-β activation in 6-OHDA-lesioned mouse model of Parkinson’s disease. CNS Neurosci. Ther..

[30] Porrini V., Mota M., Parrella E., Bellucci A., Benarese M., Faggi L., Tonin P., Spano P.F., Pizzi M. (2017). Mild Inflammatory Profile without Gliosis in the c-Rel Deficient Mouse Modeling a Late-Onset Parkinsonism. Front. Aging Neurosci..

[31] Li Y., Xu H., Wang H., Yang K., Luan J., Wang S. (2023). TREM2: Potential therapeutic targeting of microglia for Alzheimer’s disease. Biomed. Pharmacother..

[32] Liu Z., Ning J., Zheng X., Meng J., Han L., Zheng H., Zhong L., Chen X.-F., Zhang X., Luo H. (2020). TMEM59 interacts with TREM2 and modulates TREM2-dependent microglial activities. Cell Death Dis..

[33] Chen Y., Chen X., Guo X., Song W., Cao B., Wei Q., Ou R., Zhao B., Shang H.-F. (2015). Assessment of TREM2 rs75932628 association with Parkinson’s disease and multiple system atrophy in a Chinese population. Neurol. Sci..

[34] Dardiotis E., Rikos D., Siokas V., Aloizou A.M., Tsouris Z., Sakalakis E., Brotis A.G., Bogdanos D.P., Hadjigeorgiou G.M. (2021). Assessment of TREM2 rs75932628 variant’s association with Parkinson’s disease in a Greek population and Meta-analysis of current data. Int. J. Neurosci..

[35] Mengel D., Thelen M., Balzer-Geldsetzer M., Söling C., Bach J.P., Schaeffer E., Herold C., Becker T., Liepelt I., Becker J. (2016). TREM2 rare variant p.R47H is not associated with Parkinson’s disease. Park. Relat. Disord..

[36] Mo M., Tang Y., Wei L., Qiu J., Peng G., Lin Y., Zhou M., Dai W., Zhang Z., Chen X. (2021). Soluble Triggering Receptor Expressed on Myeloid Cells 2 From Cerebrospinal Fluid in Sleep Disorders Related to Parkinson’s Disease. Front. Aging Neurosci..

[37] Peng G., Qiu J., Liu H., Zhou M., Huang S., Guo W., Lin Y., Chen X., Li Z., Li G. (2020). Analysis of Cerebrospinal Fluid Soluble TREM2 and Polymorphisms in Sporadic Parkinson’s Disease in a Chinese Population. J. Mol. Neurosci..

[38] Qin Q., Wan H., Wang D., Li J., Qu Y., Zhao J., Li J., Xue Z. (2022). The Association of CSF sTREM2 With Cognitive Decline and Its Dynamic Change in Parkinson’s Disease: Analysis of the PPMI Cohort. Front. Aging Neurosci..

[39] Wijeyekoon R.S., Kronenberg-Versteeg D., Scott K.M., Hayat S., Kuan W.L., Evans J.R., Breen D.P., Cummins G., Jones J.L., Clatworthy M.R. (2020). Peripheral innate immune and bacterial signals relate to clinical heterogeneity in Parkinson’s disease. Brain Behav. Immun..

[40] Zhang X., Zhong X., Wang L., Li H., Yang L., Li X., Yu X., Xie A. (2023). Effects of soluble TREM2 on motor progression in Parkinson’s disease. Neurosci. Lett..

[41] Pagan F.L., Hebron M.L., Wilmarth B., Torres-Yaghi Y., Lawler A., Mundel E.E., Yusuf N., Starr N.J., Arellano J., Howard H.H. (2019). Pharmacokinetics and pharmacodynamics of a single dose Nilotinib in individuals with Parkinson’s disease. Pharmacol. Res. Perspect..

[42] Johnston K.G., Berackey B.T., Tran K.M., Gelber A., Yu Z., MacGregor G.R., Mukamel E.A., Tan Z., Green K.N., Xu X. (2024). Single-cell spatial transcriptomics reveals distinct patterns of dysregulation in non-neuronal and neuronal cells induced by the Trem2R47H Alzheimer’s risk gene mutation. Mol. Psychiatry.

[43] Maass F., Canaslan S., van Riesen C., Hermann P., Schmitz M., Schulte C., Brockmann K., Synofzik M., Bähr M., Zerr I. (2024). Myelin basic protein and TREM2 quantification in the CSF of patients with Multiple System Atrophy and other Parkinsonian conditions. J. Neurol..

[44] Azzolini F., Gilio L., Pavone L., Iezzi E., Dolcetti E., Bruno A., Buttari F., Musella A., Mandolesi G., Guadalupi L. (2022). Neuroinflammation Is Associated with GFAP and sTREM2 Levels in Multiple Sclerosis. Biomolecules.

[45] Li L., Xu N., He Y., Tang M., Yang B., Du J., Chen L., Mao X., Song B., Hua Z. (2025). Dehydroervatamine as a promising novel TREM2 agonist, attenuates neuroinflammation. Neurotherapeutics.

[46] Long H., Simmons A., Mayorga A., Burgess B., Nguyen T., Budda B., Rychkova A., Rhinn H., Tassi I., Ward M. (2024). Preclinical and first-in-human evaluation of AL002, a novel TREM2 agonistic antibody for Alzheimer’s disease. Alzheimers Res. Ther..

[47] Magro D., Venezia M., Rita Balistreri C. (2024). The omics technologies and liquid biopsies: Advantages, limitations, applications. Med. Omics.

